# The Effect of Laser Parameters on Cutting Metallic Materials

**DOI:** 10.3390/ma13204596

**Published:** 2020-10-15

**Authors:** Seungik Son, Dongkyoung Lee

**Affiliations:** 1 Department of Future Convergence Engineering, Kongju National University, Cheonan 1223-24, Korea; thstmddlr23@gmail.com; 2 Department of Mechanical and Automotive Engineering, Kongju National University, Cheonan 1223-24, Korea

**Keywords:** high-power laser cutting, laser parameters, cutting quality, ANOVA, multiple regression

## Abstract

This experimental study investigated the effect of laser parameters on the machining of SS41 and SUS304. The metallic materials play an important role in engineering applications. They are widely used in high-tech industries such as aerospace, automotive, and architecture. Due to the development of technology and high-tech industrialization, the various processing technologies are being developed with the requirement of high precision. However, the conventional cutting process is difficult to meet high precision processing. Therefore, to achieve high precision processing of the SS41 and SUS304, laser manufacturing has been applied. The current study investigated the process quality of laser cutting for SS41 and SUS304, with the usage of a continuous wave CO_2_ laser cutting system. The experimental variables are set to the laser cutting speed, laser power, and different engineering materials. The results are significantly affected by the laser parameters. As the result, the process quality of the laser cutting has been observed by measuring the top and bottom kerf widths, as well as the size of the melting zone and Heat Affected Zone (HAZ) according to volume energy. In addition, the evaluation of the laser processing parameters is significantly important to achieve optimal cutting quality. Therefore, we observed the correlation between the laser parameters and cutting quality. These were evaluated by analysis of variance (ANOVA) and multiple regression analysis. The experimental results of kerf top, kerf bottom, melting width, and HAZ on the laser parameters are properly predicted by multiple regression. In addition, the effect of laser parameters on the materials is determinant by the percentage of contribution of ANOVA.

## 1. Introduction

There are various metallic materials used for production in the industrial fields. Among the metallic materials commonly used in industry, SS41 and SUS304 are the most widely used. SS41 is a structural steel containing Si and Mn. It is widely used in various fields such as aerospace, automobiles, ships, and construction due to its great mechanical properties and low cost. SUS304 is stainless steel that has high corrosion resistance due to containing Cr component. It is generally used for various applications without surface treatment because the metallic materials have low thermal deformation. It is challenging to machine SS41 and SUS304 with high precision using conventional techniques such as mechanical cutting, drilling milling. The features of the mechanical method have critical processing problems such as tools wearing [1]. However, the limitation of mechanical processing can be solved by laser processing. Thus, the laser machining using CO_2_ laser is used as an alternative to the conventional method. Furthermore, the manufacturer prefers to use high-power laser processing rather than mechanical processing because the laser processing has more advantages than mechanical processing. Laser machining can be performed on various materials without tool wear and additional cost. The method is non-contact processing, which provides flexibility in processing [2,3,4,5,6,7,8,9,10,11,12,13,14,15]. Among the laser system used by industries, the CO_2_ laser has more economical than other laser systems. In addition, the laser system has high stability during the cutting or drilling processes of the significantly thick materials. Even non-metallic materials can be easily processed using a CO_2_ laser. The special concern for manufacturers using laser cutting is to maximize productivity with the high quality of components produced through the high-power laser cutting process. However, to improve product quality and productivity, the effects of laser parameters on the material should be considered as major issues. In addition, to control the influence of the laser beam, the laser parameters must be selected appropriately. Indeed, adjustable laser parameters include laser power, cutting speed, assist gas pressure, and stand-off distance. 

To maintain high precision and good quality process, the laser parameters applied to the process should be properly selected, but the effect of the parameters is difficult to predict. Besides, many manufacturers spend a lot of time and effort to determine the laser parameter which suitable for the process. In the previous studies, experiments were carried out according to specific laser parameters, and there was a comparative analysis of the effect of each parameter on the processing quality. Lamikiz et al. [16] suggested the optimum working areas and cutting conditions for the laser cutting of steel. The main experimental parameter was the thickness of the material and the results showed a remarkable different behavior between the thinnest and the thickest sheets. Kaebernick et. al. [17] described a monitoring technique in the laser cutting. The analytical techniques proved that the surface roughness was improved by controlling laser pulses. Rajaram et. al. [18] studied the effect of parameters on the characteristics of steel specimens. The material was cut through a CO_2_ laser cutting system and cutting results were analyzed with kerf width, surface roughness, and heat-affected zone. The material which was cut using the CO_2_ laser showed different results depending on the change of parameters. Yilbas [19] suggested that various parameters were affected during the laser cutting process and then, the laser power and the cutting speed for the kerf width were examined. It was confirmed that the kerf width increased with the combination of the laser power and the energy coupling factor. Anghel et. al. [20] demonstrated the experiment of laser cutting on 304 stainless steel miniature gear. In the experiment, the CO_2_ laser system was employed to cut the miniature. The effects of laser parameters on average surface roughness (R_a_) had been investigated on the surface of craters and cracks. 

The previous studies have done significant investigations on the influence of laser parameters in the laser cutting process to materials. However, there is a lack of experimental studies on comparing laser cutting of SS41 and SUS304 under different laser parameters. In this study, we studied the effect of high-power laser parameters on the different metallic materials. Multiple regression and analysis of variance (ANOVA) are used to predict the kerf width, melting width, and Heat Affected Zones (HAZ) generated after laser cutting. In addition, these are used to investigate the effects of parameter and interaction between parameters. In this paper, we firstly describe the material properties, experimental equipment, and laser parameters. Then, the experimental results are discussed. Finally, conclusions are summarized. 

## 2. Experimental Setup and Materials

**Figure 1:**
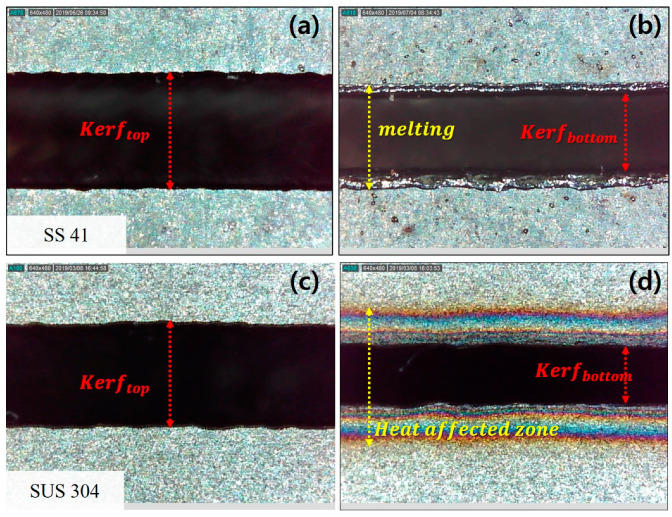
Measurement method of SS41 and SUS304 after laser cutting (**a**) top surface (**b**) bottom surface (**c**) top surface (**d**) bottom surface.

In the present study, a continuous wavelength CO_2_ laser system, which has a maximum laser power of 4.4 kW (Bylaser 4400, Bystronic, Niederönz, Switzerland), was used for the cutting process. During the experiment, the stand-off distance of the laser is set to constant, and the spot diameter is fixed at 2 mm. In addition, the laser cutting process depends on assistance gases. The assistance gases, N_2_ and O_2_, are common assistance gasses used for laser cutting on stainless steel or carbon steel [21,22]. When cutting with O_2_ gas, in the case of SS41, it is easily heated up to vaporizing temperature, thus, the material is also easily cut by a laser beam. In addition, when the SUS304 is processed using N_2_ gas, the oxidation can be protected during laser cutting. At the cutting process of the SS41 and SUS304, the assistance gases are used by the constant pressure of O_2_ and N_2_, respectively, to maintain high processing quality. Table 1 shows the laser parameters applied to SS41 and SUS304. Different laser powers and cutting speeds were conducted to cut the materials in the experiment. The laser parameters are set in the range where the material was completely cut. Table 2 shows the chemical composition of the materials used in the experiment. In order to analyze the experimental results, the kerf widths generated after the cutting process are measured on both top and bottom surfaces [23]. In addition, melting width and Heat Affected Zone (HAZ) formed in the bottom surface of the materials are measured using an optical microscope (Dino-lite AM4013MZT4, AnMo Electronics Corporation, New Taipei, Taiwan). The schematics of the kerf widths, melting width, and HAZ are shown in Figure 1. The kerf widths are the part where the laser is irradiated, and the material is completely cut-off. The kerf widths are measured in the kerf top and kerf bottom. The melting width is defined by the width of the materials with melting marks as in Figure 1 HAZ is the region where the microstructure of the materials has changed.

## 3. Results and Discussion

### 3.1. Analysis of Kerf Width in SS41 According to Volume Energy

The experimental results of laser cutting on metallic materials (SS41 and SUS304) are investigated. The kerf width of the top and bottom surface, melting width, and HAZ are analyzed according to volume energy. Volume energy is also an important parameter in the laser cutting process which is used to understand the interaction between laser and materials [24]. The volume energy ($E_{\mathrm{volume}}$) is a parameter that represents the irradiated laser per unit volume, and it is calculated by the laser power divided by the laser scanning speed and the laser beam size.

(1) $$E_{\mathrm{volume}}=\frac{P_{\mathrm{laser}}}{\mathrm{Vs}\times A}\;\left(J/m^{3}\right),$$

where $P_{\mathrm{laser}}$ is the laser power [W], $V_{s}$ is the cutting speed [mm/min], and A is the spot area of the laser beam [mm]. Experimental results are analyzed through $E_{\mathrm{volume}}$ to identify the effect on the laser powers and cutting speed.

**Figure 2:**
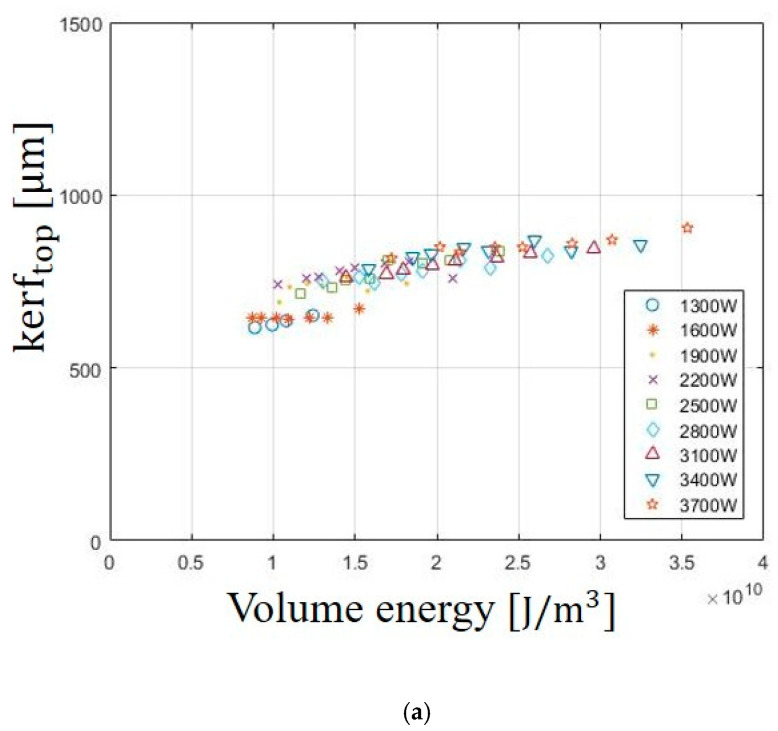

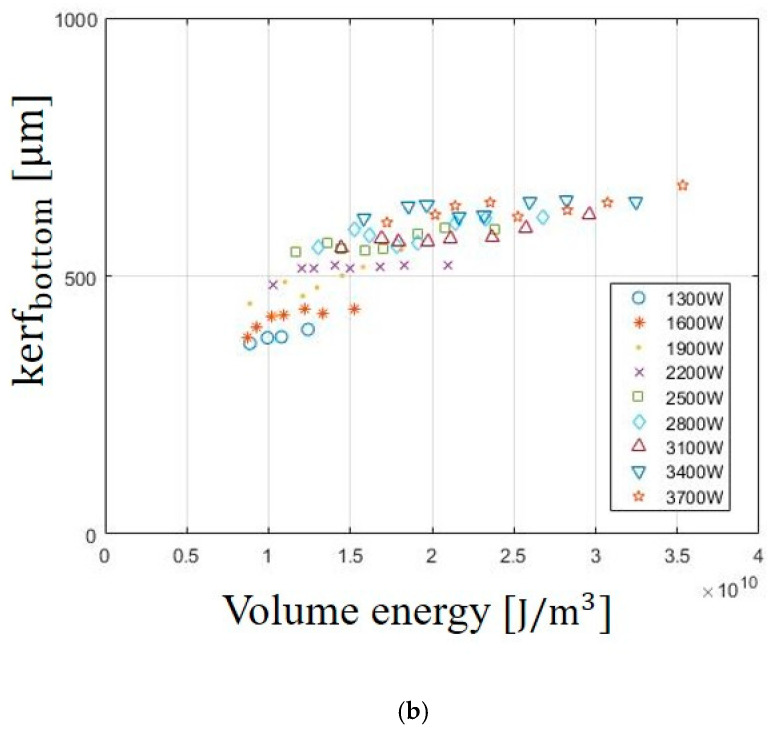
Variation of (**a**) ${\mathrm{kerf}}_{\mathrm{top}}$ and (**b**) ${\mathrm{kerf}}_{\mathrm{bottom}}$ in SS41 according to $E_{\mathrm{volume}}.$

The effect of $E_{\mathrm{volume}}$ on the kerf widths of the top and bottom surface is shown in Figure 2. The measurements of the kerf widths are conducted on both top and bottom sections of the cutting material. Each data point represents the different laser power and is obtained by averaging all measured data. The kerf widths of the top and bottom surface increase with increasing $E_{\mathrm{volume}}$. Generally, the measured kerf widths on the top surface are slightly larger than those on the bottom surface. This happens due to various reasons, such as loss of intensity of the beam, defocusing of the laser beam, or loss of gas pressure. In addition, the kerf widths of the top and bottom surface increase with increasing laser power. At the laser power of 3700 W, the kerf widths of the top and bottom surface are observed with the largest widths of 905 μm and 675 μm, respectively. In the interaction between laser and materials, it is evident that kerf widths are affected by $E_{\mathrm{volume}}$. As the $E_{\mathrm{volume}}$ increases, the material is rapidly heated. In addition, the materials are evaporated and removed easily on the top surface. Therefore, a larger kerf width of the top surface is formed than the kerf width of the bottom surface.

### 3.2. Analysis of Melting Width in SS41 According to Volume Energy

**Figure 3:**
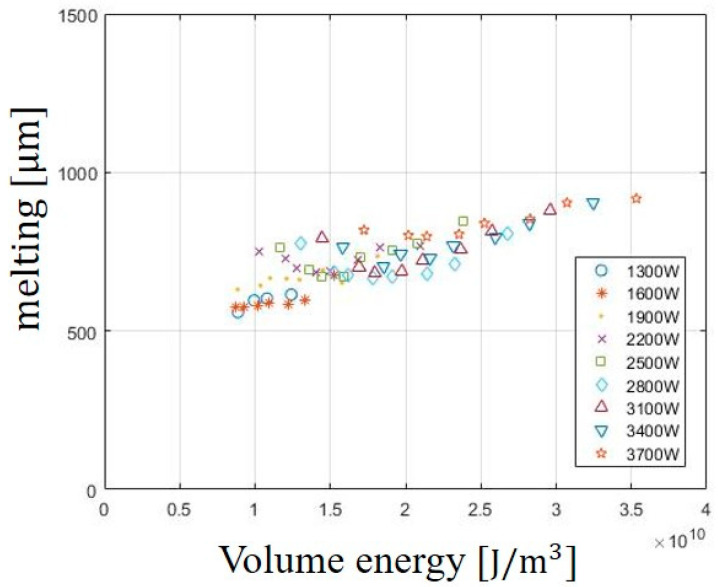
Variation of melting width in SS41 according to $E_{\mathrm{volume}}.$

The effect of $E_{\mathrm{volume}}$ on the melting width of the bottom surface is shown in Figure 3. Each measured data is obtained by averaging melting width. Melting is the area where the material melts due to the laser irradiation, and melting occurs around the kerf width. At most of the laser powers applied in the experiment, melting width increases with increasing $E_{\mathrm{volume}}$. At the laser power of 3700 W, the melting width is observed with the largest width of 917 μm. The $E_{\mathrm{volume}}$ is directly proportional to laser power. As the laser power increases, the thermal energy entering the materials increases so the melting width is observed with the largest value. In short, the laser beam including the laser power and cutting speed directly affect the material. 

### 3.3. Analysis of Kerf Width in SUS304 According to Volume Energy

**Figure 4:**
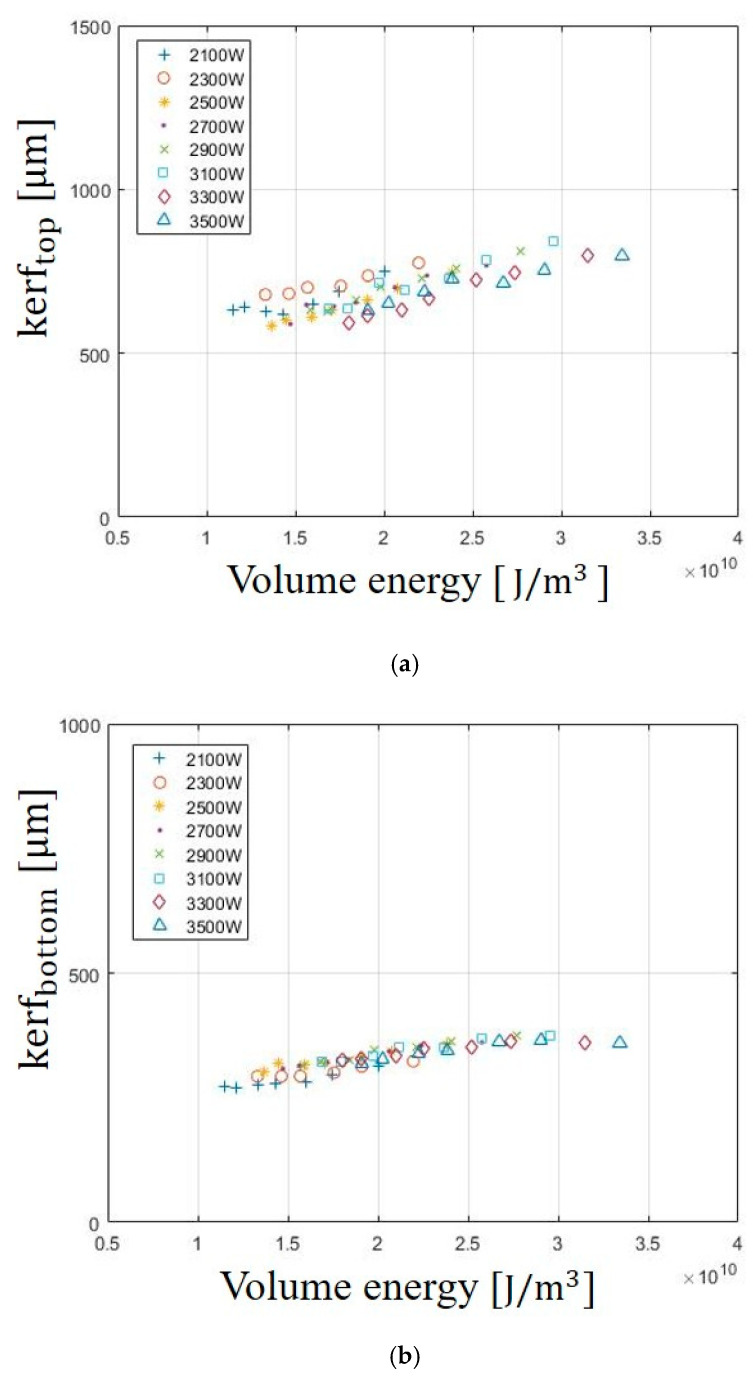
Variation of (**a**) kerf_top_ and (**b**) kerf_bottom_ in SUS304 according to E_Volume_.

The effect of $E_{\mathrm{volume}}$ on the kerf widths of the top and bottom surface are shown in Figure 4. The kerf width on SUS304 is measured in the same method as SS 41. The kerf widths of the top and bottom surface also increase with increasing $E_{\mathrm{volume}}$. The kerf widths on the top surface are slightly larger than those on the bottom surface. At the laser power of 3100 W, the kerf widths of top and bottom are observed with the largest width of 796 μm and 375 μm, respectively. As mentioned, the difference between top and bottom can be caused by various factors, such as loss of intensity of the beam, defocusing of the laser beam, or loss of gas pressure for the thickness of the materials. In the case of the trend on kerf widths, kerf widths of the top and bottom surface are observed to increase with increasing $E_{\mathrm{volume}}$. The specimen is heavily influenced by the laser beam and rapidly heats up to the vaporization temperature of the material. As the laser power increases, the laser beam entering the material increases so the kerf widths of the top and bottom surface also increase. 

### 3.4. Analysis of Heat Affected Zone in SUS304 According to Volume Energy

**Figure 5:**
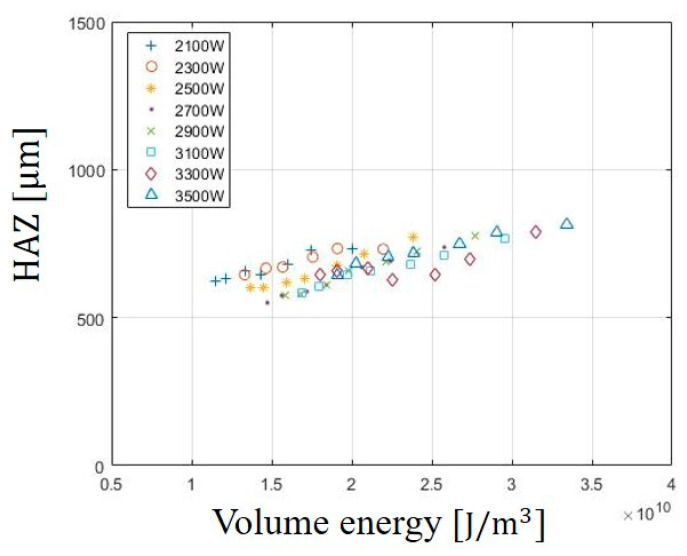
Variation of the Heat Affected Zone (HAZ) in SUS304 according to E_Volume_.

The effect of the E_Volume_ on HAZ is shown in Figure 5. HAZ is the area in which the microstructure of a material is changed by heat input. If the microstructure changes, a microcrack occurs in the processed material, it causes a partial breakdown of the product and deteriorates the quality. Therefore, it is important to reduce the HAZ during the laser cutting so that micro-cracks can be avoided. As observed from the experimental results, the effect of the E_Volume_ on the HAZ also increases with increasing E_Volume_. The maximum width of HAZ is 800 μm at 3500W and the minimum width of the HAZ is 550 μm at 2100 W. This can be related to the heat input entering the material. E_Volume_ is proportional to laser power. As the laser power increases, the heat entering materials increase and the spread of heat damage also increase. Therefore, the HAZ increases with increasing laser power.

The effect of the E_Volume_ on HAZ is shown in Figure 5. HAZ is the area in which the microstructure of a material is changed by heat input. If the microstructure changes, a microcrack occurs in the processed material, it causes a partial breakdown of the product and deteriorates the quality. Therefore, it is important to reduce the HAZ during the laser cutting so that micro-cracks can be avoided. As observed from the experimental results, the effect of the E_Volume_ on the HAZ also increases with increasing E_Volume_. The maximum width of HAZ was 800 μm at 3500 W and the minimum width of the HAZ was 550 μm at 2100 W. This can be related to the heat input entering the material. E_Volume_ is proportional to laser power. As the laser power increases, the heat entering materials increase and the spread of heat damage also increase. Therefore, the HAZ increases with increasing laser power.

### 3.5. Multiple Regression

In this section, the regression analysis of laser power and cutting speed in the laser cutting process is performed. Multiple regression analysis is a mathematical model for indicating the suitability of the relationship between the independent and dependent variable [25]. In the case of the regression model, if the high order equation is used regardless of experiment data, the determination coefficient always increases. This problem is called “overfitting”. If the regression model becomes overfitting, the prediction of experimental results through the regression model becomes meaningless. Thus, the regression equation used in this study is the quadratic regression model and the equation for the regression model is followed by:

(2) $$y=\beta_{0}+\overset{n}{\underset{i=1}{\sum }}\beta_{i}X_{i}+\overset{n}{\underset{i=1}{\sum }}\beta_{\mathrm{ii}}X_{i}^{2}+\overset{n}{\underset{i<j}{\sum }}\beta_{\mathrm{ij}}X_{i}X_{j},$$

where β is the regression coefficient and can be calculated using the least-squares method, $X_{i}$ and $X_{j}$ are the independent variables of this regression equation and these are laser power and cutting speed, respectively, y is the dependent variable and represents measured data. The second-order regression model has been developed for kerf top width, kerf bottom width, melting width, and HAZ using data from the experiments. To calculate the regression coefficient β, the coefficients of the quadratic regression model are calculated. In addition, the determination coefficient ($R_{\mathrm{sq}}$-value) and the adjusted determination coefficient ($R_{\mathrm{adj}}$) are calculated to check whether the data predicted by the regression model is appropriate. When the determination coefficient is close to 1, the accuracy of regression model is estimated to be suitable. The regression coefficients are determined by the t-test. The ‘SE Coef’ represents the standard error of the coefficient, and it is useful for making up a confidence interval and performing a hypothesis test. The t-test is a statistical method of the standardized value which is calculated from experimental data. The T-statistic is used to measure the magnitude of variation for the experimental data. It is calculated from experimental data to compare the null hypothesis. Each term of coefficients is tested by the null hypothesis according to the *p*-value. The null hypothesis is statistical proof to determine that the regression model is statistically significant. It can be determined by statistical evidence when the experimental data is meaningful. In general, a low *p*-value (<0.05) indicates that the predicted model can be meaningful in the experimental data. The regression coefficient suitability and coefficient of determination are shown in Table 3 and Table 4.

**Figure 6:**
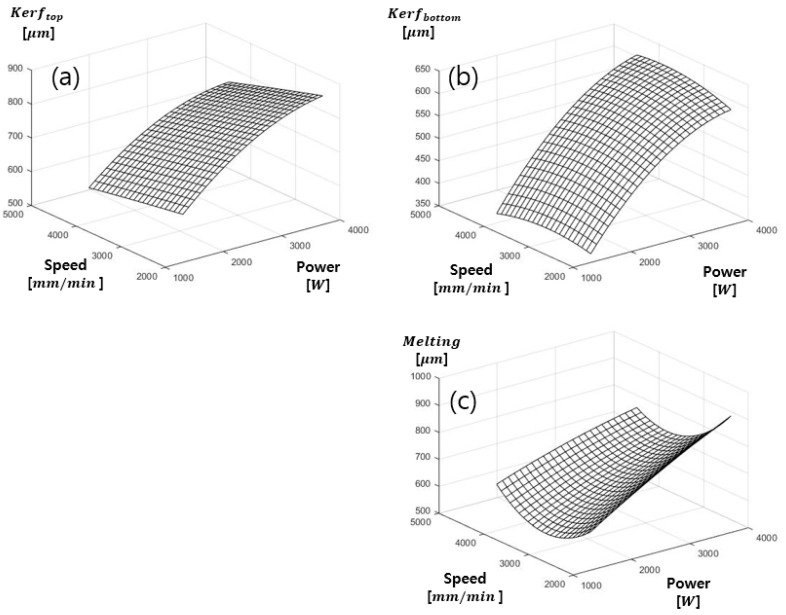
Multiple regression of SS41 (**a**) kerf_top_, (**b**) kerf_bottom_, (**c**) Melting.

The results based on the regression model for kerf widths of top, bottom surface, and melting width on SS41 on the laser power and cutting speed are plotted in Figure 6 and mathematical equations are expressed in Equations (3)–(5), respectively. The regression model of ${\mathrm{kerf}}_{\mathrm{top}}$ is shown in Figure 6a $R_{\mathrm{sq}}$ and $R_{\mathrm{sq}}$(adj) of the kerf top are 0.90 and 0.89, respectively. When the determination coefficient is close to 1, the accuracy of the regression model is high. Therefore, the experimental data are suitable for the regression model. It also shows the most appropriate coefficient of determination among the regression models. In Figure 6, it is found that the ${\mathrm{kerf}}_{\mathrm{top}}$ increases as increasing laser power. On the other hand, the variation of the ${\mathrm{kerf}}_{\mathrm{top}}$ is insignificant when the cutting speed increases. However, the ${\mathrm{kerf}}_{\mathrm{top}}$ increases when the laser power and cutting speed increase simultaneously. The regression model of ${\mathrm{kerf}}_{\mathrm{bottom}}$ is shown in Figure 6b. $R_{\mathrm{sq}}$ and $R_{\mathrm{sq}}$(adj) of ${\mathrm{kerf}}_{\mathrm{bottom}}$ are 0.89 and 0.88, respectively. This regression model is appropriate for the experimental data. It is also found that ${\mathrm{kerf}}_{\mathrm{bottom}}$ increase as the increasing laser power. However, the variation of ${\mathrm{kerf}}_{\mathrm{bottom}}$ is not variation when the cutting speed increases. It is also that the ${\mathrm{kerf}}_{\mathrm{top}}$ increases when the laser power and cutting speed increase simultaneously. This is similar to the experimental result of ${\mathrm{kerf}}_{\mathrm{top}}$. The regression model for melting width is shown in Figure 6c. The correlation model is suitable for experimental data. $R_{\mathrm{sq}}$ and $R_{\mathrm{sq}}$(adj) were 0.86 and 0.85, respectively. This leads to the fact that the data used in the regression model were well-fitted. In the relationship of the laser parameters, the melting width increases as the increasing laser power. However, the melting width first decreases when cutting speed increases up to 3000 mm/min. After the cutting speed of 3000 m/mm, the melting width increases when the cutting speed increases. In addition, when the laser power and cutting speed increase simultaneously, the melting width increases.

(3) $$\begin{array}{cc} {\mathrm{kerf}}_{\mathrm{top}} & =388.6832+0.2657X_{1}-0.00855X_{2}-3\times 10^{-5}X_{1}\times X_{1}\\ & -8.5\times 10^{-7}X_{2}\times X_{2}-7.6\times 10^{-6}X_{1}\times {\;X}_{2} \end{array}$$

(4) $$\begin{array}{cc} {\mathrm{kerf}}_{\mathrm{bottom}}= & -5.9335+\;0.2503X_{1}-0.0723X_{2}-\left(3.289\times 10^{-5}\right)X_{1}\times X_{1}-\\ & \left(1.372\times 10^{-5}\right)X_{2}\times X_{2}-\left(6.355\times 10^{-6}\right)X_{1}\times X_{2} \end{array}$$

(5) $$\begin{array}{cc} \mathrm{Melting}= & 1030.875+\;0.2174X_{1}-0.4547X_{2}-\left(7.995\times 10^{-6}\right)X_{1}\times X_{1}-\\ & \left(8.1839\times 10^{-5}\right)X_{2}\times X_{2}-\left(2.8654\times 10^{-5}\right)X_{1}\times X_{2} \end{array}$$

(6) $${\mathrm{kerf}}_{\mathrm{top}}=:853.0468+0.167979X_{1}-0.21867X_{2}-1.1\times 10^{-6}X_{1}\times X_{1}-\;4.6\times 10^{-5}X_{2}\times X_{2}-5\times 10^{-5}X_{1}\times X_{2}$$

(7) $${\mathrm{kerf}}_{\mathrm{top}}=:853.0468+0.167979X_{1}-0.21867X_{2}-1.1\times 10^{-6}X_{1}\times X_{1}-\;4.6\times 10^{-5}X_{2}\times X_{2}-5\times 10^{-5}X_{1}\times X_{2}$$

(8) $$\mathrm{Heat}\;\mathrm{Affected}\;\mathrm{Zone}=2289.716-0.68795X_{1}-0.40922X_{2}-0.000132X_{1}\times X_{1}-\;6.28\times 10^{-5}X_{2}\times X_{2}-1.4\times 10^{-5}X_{1}\times X_{2}$$

**Figure 7:**
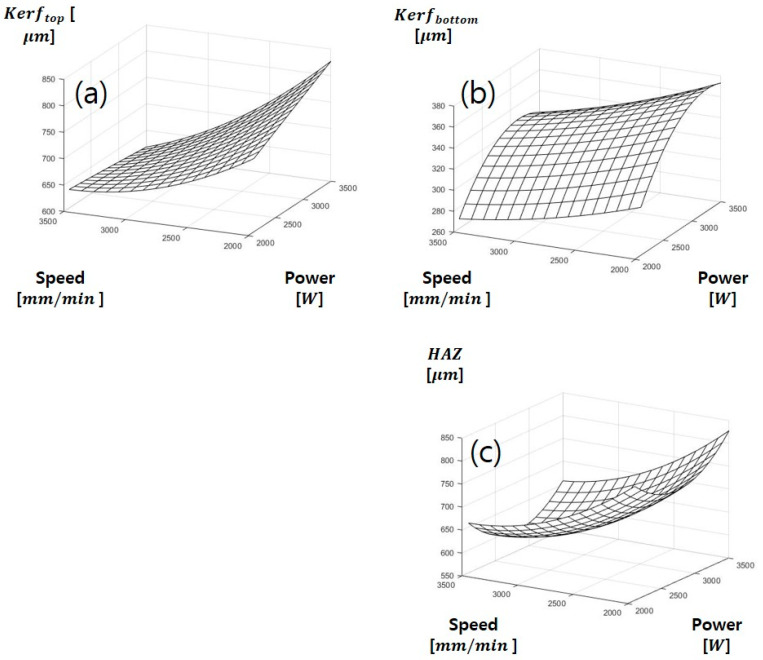
Multiple regress of SUS304 (**a**) kerf_top_, (**b**) kerf_bottom_, (**c**) Heat Affected Zone.

The regression model for kerf widths and HAZ on SUS304 is shown in Figure 7. The regression model of ${\mathrm{kerf}}_{\mathrm{top}}$ is shown in Figure 7a and mathematical equations are expressed in Equations (6)–(8), respectively. $R_{\mathrm{sq}}$ and $R_{\mathrm{sq}}$(adj) are 0.80 and 0.78, respectively. The regression model is relatively suitable for experimental data. In the relation between laser power and cutting speed, it is found that the ${\mathrm{kerf}}_{\mathrm{top}}$ increases as the decreasing cutting speed but the variation of the ${\mathrm{kerf}}_{\mathrm{top}}$ is insignificant when the laser power increase. When the laser power and cutting speed increase simultaneously the variation of ${\mathrm{kerf}}_{\mathrm{top}}$ is relatively low. The regression model of ${\mathrm{kerf}}_{\mathrm{bottom}}$ is shown in Figure 7b. $R_{\mathrm{sq}}$ and $R_{\mathrm{sq}}$(adj) of ${\mathrm{kerf}}_{\mathrm{bottom}}$ are 0.92 and 0.91, respectively. The experimental data are suitable for the regression model. It is also the most appropriate decision coefficient among the regression models for SUS304. In the effects of laser power and cutting speed on ${\mathrm{kerf}}_{\mathrm{bottom}},$ it is also found that ${\mathrm{kerf}}_{\mathrm{bottom}}$ increases as the increasing laser power. However, there is a little variation of the ${\mathrm{kerf}}_{\mathrm{bottom}}$ when the cutting speed increases. When the laser cutting speed increases up to 35,000 mm/min and the laser power increases up to 3000 W, the ${\mathrm{kerf}}_{\mathrm{bottom}}$ increases but, after 3000 W laser power, then it decreases slightly. The regression model for HAZ is shown in Figure 7c. $R_{\mathrm{sq}}$ and $R_{\mathrm{sq}}$(adj) are 0.85 and 0.83, respectively. This regression is in good agreement with the experimental data. In the relationship of the laser parameters, as the cutting speed increases, HAZ decreases rapidly. In addition, HAZ first decreases when laser power increases up to 2500 W but after laser power of 2500 W the HAZ increases with increasing the laser power. 

### 3.6. Analysis of Variance (ANOVA)

In this section, the effect of the laser parameter is investigated through the analysis of variance (ANOVA). The ANOVA statistically analyzes the effect of each independent variable on the dependent variable during laser cutting. The advantage of ANOVA can be identified by the important factors for each independent variable, as well as the interaction effect of each parameter on laser cutting quality [26]. The variability of the experimental data can be determined by the percentage of contribution (PCR) of each independent variable. In addition, the results of the ANOVA are represented by the 95% confidence level (*p* ≤ 0.05) and it is considered that the independent variable has a statistically significant effect on the experimental data. Table 5 and Table 6 for ANOVA results show Degrees of Freedom (DF), Sum of Squares (SS), Mean squares (MS), F ratio, and percentage of contribution (PCR). The SS is the sum of the squared deviations between the mean and the variance of each experimental data. The MS represents the estimate of the population variance. This is the corresponding sum of squares divided by degrees of freedom. The F ratio is the distribution ratio obtained through a comparison of variances. It is used to test whether the variance of each group is different and whether the population mean is different. The PCR is calculated based on the estimated variance components. The higher PCR indicates that the variability of the experimental data by independent variables increases. In the results of ANOVA, the P-value on the effect of each parameter and interaction effects between parameters are less than 0.05. This indicates that the parameters used have a significant effect on the experimental results. 

The ANOVA results for SS41 are shown in Table 5. ANOVA tables demonstrate the results of laser power, cutting speed, and laser power × cutting speed for the 95% confidence level (*p* < 0.05). At the ANOVA table of ${\mathrm{kerf}}_{\mathrm{top}}$, it shows that the most effective variable is laser power which was 59.28% of the PCR. The other variables affecting ${\mathrm{kerf}}_{\mathrm{top}}$ were cutting speed and laser power × cutting speed, which were 12.48% and 27.99% of PCR, respectively. At the ANOVA table of the ${\mathrm{kerf}}_{\mathrm{bottom}}$, the laser power was the most effective variable, which was 73.06% of PCR. The other variables affecting ${\mathrm{kerf}}_{\mathrm{bottom}}$ were cutting speed and laser power × cutting speed, which were 5.63% and 20.37% of PCR, respectively. As a result of melting width, the PCR of the laser power, cutting speed, and laser power × cutting speed were found to be 59.65%, 12.08%, and 27.35%, respectively. The ANOVA results for SUS304 are shown in Table 6. At the ANOVA table of ${\mathrm{kerf}}_{\mathrm{top}}$, it shows that the most effective variable is laser power × cutting speed which was 78.33% of the PCR. The other variables affecting ${\mathrm{kerf}}_{\mathrm{top}}$ were laser power and cutting speed which were 9.93% and 10.45% of PCR, respectively. As the results of ${\mathrm{kerf}}_{\mathrm{bottom}}$, it shows that the most effective variable was laser power × cutting speed, which was 40.25% of PCR. The other variables affecting ${\mathrm{kerf}}_{\mathrm{bottom}}$ were laser power and cutting speed which were 38.3% and 20.22% of PCR, respectively. At the ANOVA results of HAZ, the PCR of the laser power, cutting speed, and laser power × cutting speed were found to be 22.39%, 28.74%, and 40.78%, respectively. In the case of SS41 analyzed by ANOVA, the most effective variable of ${\mathrm{kerf}}_{\mathrm{top}}$, ${\mathrm{kerf}}_{\mathrm{bottom}}$, and melting was laser power. On the other hand, at the ANOVA results of SUS304, the most effective variable of the ${\mathrm{kerf}}_{\mathrm{top}}$ was laser power and the most effective variables of ${\mathrm{kerf}}_{\mathrm{bottom}}$ and HAZ was laser power × cutting speed. The most effective variables of experimental results were different. The reason why the effective variable is different is the mechanical or chemical properties of metallic materials are different. In the case of the chemical properties of materials, SUS304 includes the chemical composition of Ni and Cr. These components improve corrosion resistance and heat resistance. Especially, The Cr component interacts with the atmosphere of the O and then, the thin film is generated on the SUS304 surface [27]. This thin film can protect from the surface corrosion and heat damage and the effect of laser power might decrease due to the protecting thin film. Therefore, we assume that the effect of laser power affecting the material is low. The complex effect of laser power × cutting speed has more influence on the material than the effect of laser power. The influence of laser parameters on the components such Ni and Cr needs further study.

## 4. Conclusions

Nowadays, there are many types of laser systems, such as Nd:YAG laser or CO_2_ laser. The CO_2_ laser system has many advantages such as providing good processing quality and high processing efficiency [28]. To achieve improvement in product quality and productivity, the effects of laser parameters on the material should be considered as a major issue. In this study, the influences of the laser parameter, such as laser power and cutting speed on the SS41 and SUS304 are studied. The experimental results of laser cutting on metallic materials are analyzed through multiple regression and analysis of variance (ANOVA). The effects of each independent variable to output variables are analyzed. The conclusions of this experiment are as follows:

1. We confirmed that the experimental results depend on the laser parameters. For the experimental results on $E_{\mathrm{line}}$, as $E_{\mathrm{line}}$ increases, the materials are heated until they evaporate rapidly and remove material easily. Furthermore, the laser power increases, the heat entering materials increases and the spread of heat damage also increases, so the melting and HAZ width also increase.
2. In the case of multiple regression on the SSand SUS it is founded that the experimental results in kerf widths, melting, and HAZ are affected by laser parameters. The effect of laser power and cutting speed is analyzed through the multiple regression model. The regression equation can appropriately predict output variables from independent variables. Besides, the coefficient of determination ($R_{\mathrm{sq}}$) for ${\mathrm{kerf}}_{\mathrm{top}}$, ${\mathrm{kerf}}_{\mathrm{bottom}}$, and melting width for SSare and respectively. For the SUS the $R_{\mathrm{sq}}$ for ${\mathrm{kerf}}_{\mathrm{top}}$, ${\mathrm{kerf}}_{\mathrm{bottom}}$, and HAZ are and respectively. Each of $R_{\mathrm{sq}}$ is suitable for experimental data and the regression model makes it possible to predict the effect of laser parameters on the material.
3. The results of the ANOVA on the SSand SUSanalyze the effect of each independent variable on the dependent variable during laser cutting. The most effective variable in ${\mathrm{kerf}}_{\mathrm{top}}$, ${\mathrm{kerf}}_{\mathrm{bottom}}$, and melting width on SSis laser power. In the case of ${\mathrm{kerf}}_{\mathrm{top}}$ on the SUS the most effective variables are laser power × cutting speed. On the other hand, for the ${\mathrm{kerf}}_{\mathrm{bottom}}$ and HAZ, the interaction effects of the laser power × cutting speed have been found most effective variables. The most effective variables are determined differently on SS41 and SUS 304. This may be caused by different chemical properties of metallic materials. Especially, we assumed that the influence of Ni and Cr components in SUS304 plays a critical role in the laser cutting. Therefore, the effect of laser cutting parameters on the chemical properties of SUS304 needs further study.

## Figures and Tables

**Table 1:** Laser parameters.

	SS41	SUS304
Laser Power [W]	1000–3700	2100–3900
Cutting Speed [mm/s]	2000–4100	2000–3500
Assistance Gas	O_2_	N_2_
Gas Pressure [bar]	3	3
Thickness [mm]	2	2

**Table 2:** Materials chemical composition.

	C	Si	Mn	P	S	Ni	Cr
SS41 Properties [%]	0.14~0.22	≤0.3	0.36~0.65	≤0.045	≤0.05		
SUS304 Properties [%]	0.08	1.00	2.00	0.45	0.30	8.00~10.50	18.00~20.00

**Table 3:** The regression coefficient of SS41.

$Kerf_{top}.$	**Coefficient**	**SE Coefficient**	**T Statistic**	***p*-Value**
$\beta_{0}$	388.6832	80.07202	4.85417	8.75 × 10^−6^
$\beta_{1}$	0.2657	0.033199	8.00299	4.35 × 10^−11^
$\beta_{2}$	−0.00855	0.044628	−0.1916	0.848694
$\beta_{3}$	−3 × 10^−5^	6.2 × 10^−6^	−4.87501	8.11 × 10^−6^
$\beta_{4}$	−8.5 × 10^−7^	7.18 × 10^−6^	−0.1179	0.906538
$\beta_{5}$	−7.6 × 10^−6^	7.02 × 10^−6^	−1.08053	0.28416
$R_{\mathrm{sq}}$ = 0.90, $R_{\mathrm{sq}}$(adj) = 0.89				
$kerf_{bottom}$	**Coefficient**	**SE Coefficient**	**T Statistic**	***p*-Value**
$\beta_{0}$	−5.9335	89.49774223	−0.066297523	0.947357769
$\beta_{1}$	0.2503	0.037857046	6.611759502	1.0722 × 10^−8^
$\beta_{2}$	0.0723	0.05112402	1.413235703	0.16266912
$\beta_{3}$	−3.289 × 10^−5^	7.25866 × 10^−6^	−4.531773519	2.7836 × 10^−5^
$\beta_{4}$	−1.372 × 10^−5^	8.75522 × 10^−6^	−1.566708952	0.122356105
$\beta_{5}$	6.355 × 10^−6^	8.60318 × 10^−6^	0.738711082	0.462915371
$R_{\mathrm{sq}}$ = 0.89, $R_{\mathrm{sq}}$(adj) = 0.88				
Melting	**Coefficient**	**SE Coefficient**	**T Statistic**	***p*-Value**
$\beta_{0}$	1030.875	112.6616	9.150191	4.76 × 10^−13^
$\beta_{1}$	0.2174	0.046711	4.653414	1.81 × 10^−5^
$\beta_{2}$	−0.4547	0.062792	−7.24157	8.9 × 10^−10^
$\beta_{3}$	−7.995 × 10^−6^	8.73 × 10^−6^	−0.91606	0.363242
$\beta_{4}$	8.1839 × 10^−5^	1.01 × 10^−5^	8.104238	2.91 × 10^−11^
$\beta_{5}$	−2.8654 × 10^−5^	9.88 × 10^−6^	−2.8996	0.005187
$R_{\mathrm{sq}}$ = 0.86; $R_{\mathrm{sq}}$(adj) = 0.85				

**Table 4:** Regression coefficient of SUS304.

$Kerf_{top}.$	**Coefficient**	**SE Coefficient**	**T Statistic**	***p*-Value**
$\beta_{0}$	853.0468	251.3251	3.394196	0.001371
$\beta_{1}$	0.167979	0.129446	1.297679	0.200474
$\beta_{2}$	−0.21867	0.11114	−1.96752	0.054797
$\beta_{3}$	−1.1 × 10^−6^	2.14 × 10^−5^	−0.05021	0.960161
$\beta_{4}$	4.6 × 10^−5^	1.84 × 10^−5^	2.498231	0.015886
$\beta_{5}$	−5 × 10^−5^	1.76 × 10^−5^	−2.82229	0.006871
$R_{\mathrm{sq}}$ = 0.80, $R_{\mathrm{sq}}$(adj) = 0.78				
$kerf_{botom}$	**Coefficient**	**SE Coefficient**	**T Statistic**	***p*-Value**
$\beta_{0}$	−108.267	69.11328	−1.56651	0.123665
$\beta_{1}$	0.360518	0.035597	10.12777	1.32 × 10^−13^
$\beta_{2}$	−0.05506	0.030563	−1.8015	0.077778
$\beta_{3}$	−5.3 × 10^−5^	5.9 × 10^−6^	−8.97845	6.35 × 10^−12^
$\beta_{4}$	8.64 × 10^−6^	5.06 × 10^−6^	1.707061	0.094141
$\beta_{5}$	−8.1 × 10^−6^	4.83 × 10^−6^	−1.67321	0.100659
$R_{\mathrm{sq}}$ = 0.92, $R_{\mathrm{sq}}$(adj) = 0.91				
Heat Affected Zone	**Coefficient**	**SE Coefficient**	**T Statistic**	***p*-Value**
$\beta_{0}$	2289.716	218.9419	10.4581	4.46 × 10^−14^
$\beta_{1}$	−0.68795	0.112767	−6.10062	1.64 × 10^−7^
$\beta_{2}$	−0.40922	0.09682	−4.22663	0.000103
$\beta_{3}$	0.000132	1.87 × 10^−5^	7.041524	5.72 × 10^−9^
$\beta_{4}$	6.28 × 10^−5^	1.6 × 10^−5^	3.913384	0.000281
$\beta_{5}$	−1.4 × 10^−5^	1.53 × 10^−5^	−0.91761	0.363319
$R_{\mathrm{sq}}$ = 0.85, $R_{\mathrm{sq}}$(adj) = 0.83				

**Table 5:** SS41ANOVA table.

Source	SS	DF	MS	F Ratio	*p*-Value	PCR [%]
$Kerf_{top}$						
Laser Power	5.6 × 10^6^	8	7.0 × 10^5^	4.2 × 10^3^	<0.05	59.28
Cutting Speed	1.2 × 10^6^	7	1.7 × 10^5^	1.0 × 10^3^	<0.05	12.48
Laser power × Cutting speed	2.7 × 10^6^	56	4.7 × 10^4^	2.8 × 10^2^	<0.05	27.99
Error	2.4 × 10^4^	144	1.7 × 10^2^			0.26
Total	9.5 × 10^6^	215				
$Kerf_{bottom}$						
Laser Power	4.0 × 10^6^	8	5.0 × 10^5^	1.4 × 10^3^	<0.05	73.06
Cutting Speed	3.1 × 10^5^	7	4.4 × 10^4^	1.2 × 10^2^	<0.05	5.63
Laser power × Cutting speed	1.1 × 10^6^	56	2.0 × 10^4^	5.6 × 10	<0.05	20.37
Error	5.1 × 10^4^	144	3.6 × 10^2^			0.94
Total	5.5 × 10^6^	215				
Melting						
Laser Power	5.3 × 10^6^	8	6.6 × 10^5^	1.2 × 10^3^	<0.05	59.65
Cutting Speed	1.1 × 10^6^	7	1.5 × 10^5^	2.7 × 10^2^	<0.05	12.08
Laser power × Cutting speed	2.4 × 10^6^	56	4.3 × 10^4^	7.6 × 10	<0.05	27.35
Error	8.2 × 10^4^	144	5.7 × 10^2^			0.92
Total	8.9 × 10^6^	215				

**Table 6:** SUS304 ANOVA table.

Source	SS	DF	MS	F Ratio	*p*-Value	PCR [%]
$Kerf_{top}$						
Laser Power	9.0 × 10^4^	9	1.0 × 10^4^	1.2 × 10^2^	<0.05	9.93
Cutting Speed	9.5 × 10^4^	6	1.6 × 10^4^	1.9 × 10^2^	<0.05	10.45
Laser power × Cutting speed	7.1 × 10^5^	54	1.3 × 10^4^	1.6 × 10^2^	<0.05	78.33
Error	1.2 × 10^4^	140	8.4 × 10			1.29
Total	9.1 × 10^5^	209				
$Kerf_{bottom}$						
Laser Power	1.9 × 10^5^	9	2.1 × 10^4^	3.9 × 10^2^	<0.05	38.03
Cutting Speed	9.9 × 10^4^	6	1.6 × 10^4^	3.1 × 10^2^	<0.05	20.22
Laser power × Cutting speed	2.0 × 10^5^	54	3.6 × 10^3^	6.9 × 10	<0.05	40.25
Error	7.4 × 103	140	5.3 × 10			1.52
Total	4.9 × 10^5^	209				
**Heat Affected Zone**						
Laser Power	6.0 × 10^5^	9	6.7 × 10^4^	4.3 × 10	<0.05	22.39
Cutting Speed	7.7 × 10^5^	6	1.3 × 10^5^	8.3 × 10	<0.05	28.74
Laser power × Cutting speed	1.1 × 10^6^	54	2.0 × 10^4^	1.3 × 10	<0.05	40.78
Error	2.2 × 10^5^	140	1.6 × 10^3^			8.09
Total	2.7 × 10^6^	209				
